# Increased Expression of Protease-Activated Receptor 4 and Trefoil Factor 2 in Human Colorectal Cancer

**DOI:** 10.1371/journal.pone.0122678

**Published:** 2015-04-13

**Authors:** Guoyu Yu, Ping Jiang, Yang Xiang, Yong Zhang, Zhu Zhu, Chuanrao Zhang, Siman Lee, Wenhui Lee, Yun Zhang

**Affiliations:** 1 Key Laboratory of Animal Models and Human Disease Mechanisms, Kunming Institute of Zoology, Chinese Academy of Sciences, Kunming, Yunnan 650223, China; 2 Department of Biochemistry, Kunming Medical University, Kunming, Yunnan 650500, China; 3 Department of Pathology and Pathophysiology, Kunming Medical University, Kunming, Yunnan 650500, China; 4 Department of Gastroenterology, the First Affiliated Hospital of Kunming Medical University, Kunming 650032, China; 5 Department of Functional Experimental Center, Kunming Medical University, Kunming, Yunnan 650500, China; Sun Yat-sen University Medical School, CHINA

## Abstract

Protease-activated receptor 4 (PAR4), a member of G-protein coupled receptors family, was recently reported to exhibit decreased expression in gastric cancer and esophageal squamous cancer, yet increased expression during the progression of prostate cancer. Trefoil factor 2 (TFF2), a small peptide constitutively expressed in the gastric mucosa, plays a protective role in restitution of gastric mucosa. Altered TFF2 expression was also related to the development of gastrointestinal cancer. TFF2 has been verified to promote cell migration via PAR4, but the roles of PAR4 and TFF2 in the progress of colorectal cancer are still unknown. In this study, the expression level of PAR4 and TFF2 in colorectal cancer tissues was measured using real-time PCR (n = 38), western blotting (n=38) and tissue microarrays (n = 66). The mRNA and protein expression levels of PAR4 and TFF2 were remarkably increased in colorectal cancer compared with matched noncancerous tissues, especially in positive lymph node and poorly differentiated cancers. The colorectal carcinoma cell LoVo showed an increased response to TFF2 as assessed by cell invasion upon PAR4 expression. However, after intervention of PAR4 expression, PAR4 positive colorectal carcinoma cell HT-29 was less responsive to TFF2 in cell invasion. Genomic bisulfite sequencing showed the hypomethylation of PAR4 promoter in colorectal cancer tissues and the hypermethylation in the normal mucosa that suggested the low methylation of promoter was correlated to the increased PAR4 expression. Taken together, the results demonstrated that the up-regulated expression of PAR4 and TFF2 frequently occurs in colorectal cancer tissues, and that overexpression of PAR4 may be resulted from promoter hypomethylation. While TFF2 promotes invasion activity of LoVo cells overexpressing PAR4, and this effect was significantly decreased when PAR4 was knockdowned in HT-29 cells. Our findings will be helpful in further investigations into the functions and molecular mechanisms of Proteinase-activated receptors (PARs) and Trefoil factor factors (TFFs) during the progression of colorectal cancer.

## Introduction

The progression of colorectal cancer is a multistep process involved in polygenetic alterations in prooncogenes and/or tumor suppressor genes, and aberrant epigenetic gene regulation can lead to abnormal growth of malignant tumors [1,2]. PARs are seven-transmembrane G protein-coupled receptors (GPCR) comprising four members named PAR1, PAR2, PAR3, and PAR4 [3]. As having the ability of degradation of extracellular matrix proteins, PARs serve as signal molecules involved in tumor cell migration, invasion and metastasis [4]. PAR1, which was widely expressed in cancers, promoted tumor genesis and invasion of breast cancer cells and colorectal cells [5,6]. PAR2, overexpressed in prostate cancer, promoted prostate cancer cell migration [7]. On the contrary, PAR2 also showed a tumor-protective role in skin carcinogenesis [8]. PAR3 was found in kidney and liver cancer [9,10]. PAR4 expression is strongly detected in lung, thyroid, pancreas, small intestine and testis by Northern blot [11]. However, the expression and potential roles of PAR4 in tumorigenesis are still unknown. PAR4 expression was absent in normal colon mucosa, but appeared obvious staining in the dysplastic and colorectalous mucosa. PAR4 mRNA was found in 10 out of 14 (71%) human colorectal carcinoma cell lines [12].

Trefoil factors (TFFs), widely expressed in the mucosa of gastrointestinal tract, are small and compact peptides containing one or two trefoil domains. Three closely related TFFs are known in human, pS2 (TFF1), spasmolytic polypeptide (SP or TFF2), and intestinal trefoil factor (ITF or TFF3) [13]. TFFs peptides are believed to contribute to mucosal healing and restitution by virtue of promoting cell migration and suppressing apoptosis [14]. TFFs are also involved in tumorigenesis [15]. TFF2, containing two trefoil domains, is believed to be the principal cytoprotective trefoil factor in the stomach, and the expression level of TFF2 was deregulated in gastric ulcer and cancer tissue [16]. In our recent research, TFF2 has been shown to promote epithelial cell migration and wound healing via PAR4 involved phosphorylation of ERK1/2 [17], but the detail functions and molecular mechanisms of PAR4 and TFF2 in the progression of gastrointestinal tumor have not been discovered. In the study, we showed the expression levels of PAR4 and TFF2 were increased in colorectal cancer tissues when compared with the matched noncancerous mucosa, and the up-regulation expression of PAR4 in colorectal cancers may be resulted from the promoter hypomethylation. Furthermore, our data showed that TFF2 promotes colorectal cancer cell invasion by activating PAR4.

## Materials and Methods

### Ethics statement

The study was approved by Kunming Institute of Zoology, the Chinese Academy of Sciences and Kunming Medical University. Written informed consent was obtained from the patients before obtaining samples for this study.

### Study subjects

A total of 38 patients with colorectal cancer, who agreed to participate in our study, signed the informed consent form and received operations at the First Affiliated Hospital of Kunming Medical University. Colorectal specimens were obtained from colorectal tumor tissues and the adjacent non-cancer areas, which were at least 6 centimeters away from the tumor. The collected tissues were further verified by histology and were immediately frozen in liquid nitrogen and stored at -80°C until use. Colorectal cancer tissue microarray representing 66 colorectal cancers with their non-neoplastic resection margins constructed [18] were from Shanghai Outdo Biochip Center (Shanghai, China).

### RNA extraction and polymerase chain reaction (PCR)

RNA extraction and the first-strand cDNA synthesis were performed as previously described [19]. For semi-quantitative RT-PCR and real-time PCR, the primers used were as follows: the primers for PAR4 (147 bp product) were 5’-CCTTCATCTACTACTACTACGTGTCG-3’ (forward) and 5’-ACTGGAGCAAAGAGGAGTGG-3’ (reverse); for TFF2 (74 bp product) were 5’- CTGCTTCTCCAACTTCATCT-3’ (forward) and 5’-CTTAGTAATGGCAGTCTTCC-3’ (reverse); and for internal control Glyceraldehyde 3-phosphate dehydrogenase (GAPDH, 107 bp product), were 5’-ATGGGGAAGGTGAAGGTCG-3’ (forward) and 5’-GGGGTCATTGATGGCAACAATA-3’ (reverse). After RT-PCR, the amplicons of PAR4, TFF2 and GAPDH were electrophoresed in 2% agarose gels, stained by ethidium bromide and viewed under ultraviolet illumination.

Quantitative PCR was performed with a Continuous fluorescence Detector (Opticon Monitor, Bio-Rad, Hercules, CA, USA). PCR reactions for PAR4, TFF2 and GAPDH were performed using an SYBR Green real-time PCR kit (TaKaRa, Dalian, China) with the condition of: initial denaturation at 95°C for 1 min, followed by 40 cycles of 95°C for 15 s, 60°C for 15 s, and 72°C for 20 s. Each sample was run in triplicate. No template controls (no cDNA in PCR) were run to detect nonspecific or genomic amplification and primer dimerization. Fluorescence curve analysis was carried out using Opticon Monitor software. Relative quantitative evaluation of PAR4 and TFF2 expression levels were performed by E-method and expressed as a ratio of the transcript of PAR4 (TFF2) to GAPDH in the tumor tissue. The identities of RT-PCR and real-time PCR products were confirmed by DNA sequencing.

### Tissue immunohistochemistry

Tissue immunohistochemistry was performed as previously described [12]. Briefly, antigen retrieval was performed by heating in an autoclave at 121°C for 5 min. Dewaxed sections were pre-incubated with blocking serum and then incubated at 4°C overnight with the anti-human PAR4 antibody (C-20, 1:1200; Santa Cruz Biotechnology, CA, USA) and the anti-human TFF2 antibody (P-19, 1:800; Santa Cruz Biotechnology, CA, USA), respectively. Specific binding was detected by a streptavidin-biotin-peroxidase assay kit (Maxim, Fujian, China). The section was counterstained with Harris hematoxylin. Direct microscopic micrographs were captured using a Leica DFC320 camera controlled by Leica IM50 software (Leica, Germany). Sections incubated with normal goat IgG were served as a negative control. Specificity of the antibodies for PAR4 and TFF2 was confirmed by pre-incubation overnight at 4°C with their respective antigens (Santa Cruz) in a 20-fold molar excess of antigen to antibodies. Pre-incubation with PAR4 and TFF2 antigen resulted in an absence of immunolabeling.

Immunohistochemical staining was assessed semi-quantitatively by measuring both the intensity of the staining (0, 1, 2, or 3) and the extent of staining (0, 0%; 1, 0–10%; 2, 10–50%; 3, 50–100%). The scores for the intensity and extent of staining were multiplied to give a weighted score for each case (maximum possible, 9). For the statistical analysis, the weighted scores were grouped in two categories where scores of 0–3 were considered negative and 4–9 positive [20].

### Western blot

Tissue samples were homogenized in Radioimmunoprecipitaion Assay Buffer (Sigma) containing cocktail of protease inhibitors (Sigma). The protein concentration was determined by a protein assay kit (Bio-Rad). Samples (containing 30 μg of protein) were loaded on an SDS-polyacrylamide gel electrophoresis (SDS-PAGE) gel and then transferred onto a PVDF membrane. The membranes were subsequently blocked with 3% bovine serum albumin (BSA) and incubated with appropriate PAR4 and TFF2 primary and secondary antibodies, respectively. Proteins were visualized with Super Signal reagents (Pierce, Rockford, IL, USA).

### Bisulfite sequencing

Genomic DNA from colorectal cancers and non-neoplastic tissues was isolated with the Universal Genomic DNA Extraction Kit (TaKaRa) and bisulfite-converted using the CpGenome Fast DNA Modification Kit (CHEMICON). PAR4 promoter sequences were amplified from bisulfite-converted DNA by PCR, purified from agarose gels and subcloned into the pMD19-T Vector (TaKaRa). For each sample, 19 individual clones were sequenced to identify methylated cytosine residues. PCR primer sequences (forward and reverse) for PAR4 were 5’-TTTAAGGGTGATTTTAGGAAAGGTTTAGAG-3’ and 5’-ACTATAACCTCAAACTTCCTACCTC-3’.

### Cell culture

Human colorectal cancer cell lines LoVo and HT-29 were from the American Type Culture Collection (Manassas, VA, USA). The LoVo cells overexpressing PAR4 (LoVo-PAR4) and the cells transfected with a mock plasmid (LoVo-mock) were selected by 800ug/mL G418 according to our previously studies [17]. LoVo cells were cultured in Ham’s F12 medium supplemented with 10% (v/v) fetal bovine serum, 100 U/ml penicillin and 100 U/ml streptomycin, and were grown in a humidified atmosphere with 5% CO_2_ at 37°C. For treatment with 5-aza-2’-deoxycytidine (5-Aza-dC; Sigma, 95 St. Louis, MO, USA), cells were seeded at a density of 1 × 10^6^ in a 60-mm dish. After 24 h, cells were treated with 10 **μ**M of 5-Aza-dC. DMSO was treated in parallel as a control. Total cells were collected after 72 hours of addition 5-Aza-dC and subjected to RT-PCR and Western blotting analysis.

### Knockdown of PAR4 in HT-29 cells

Lentivirus expressing shRNA were generated by co-transfecting PAR4 shRNA plasmids with pCMV-dR8.2 dvpr and pCMV-VSVG packaging plasmids into 293FT cells. HT-29 cells transfected with pGIPZ-shPAR4 or empty vector pGIPZ were selected with 5 μg/ml puromycin to generate HT-29-shPAR4 and HT-29-pGIPZ, respectively. The cells were cultured in DMEM supplemented with 10% (v/v) fetal bovine serum, 100 U/ml penicillin and 100 U/ml streptomycin.

### Cell invasion

Invasion activities of LoVo-mock and LoVo-PAR4 cells, and HT-29-pGIPZ and HT-29-shPAR4 cells in vitro were measured using the Matrigel invasion assay [21]. The InnoCyte Cell Invasion Assay Kit (8 μm pore, Calbiochem, MERCK) was used according to the manufacturer’s protocol. Briefly, 5 × 10^4^ cells of LoVo-mock, LoVo-PAR4, HT-29-pGIPZ and HT-29-shPAR4 were respectively added to the upper chamber, and medium containing recombinant TFF2 was added to the lower chamber. After incubation 24 hours at 37°C, cell suspension were discarded and the upper chamber inserts were gently placed in cell staining solution for 30min. Following the lower chamber cells containing the dislodged cells were incubated an additional 30min in the same conditions. Finally 200μL the dislodged cells were transferred doubly to the wells of a 96-well plate, and measured the fluorescence at an excitation wavelength of 485±10nm and an emission wavelength of 520±10nm. Meanwhile, the lower chamber cells were photographed through the confocal laser fluorescence microscopy.

### Statistical analysis

All statistical analyses were performed by the SPSS 11.0 software (SPSS Inc., Chicago, IL, USA). The chi squared test (Tables 1 and 2) and Mann-Whitney U Text were used for the significance of correlations between PAR4 and TFF2 expression and clinical pathological parameters. Differences in the numerical data between the paired groups were evaluated using the paired Student’s test. The level of statistical significance was set at the level of *p*<0.05.

**Table 1:** Association between the mRNA levels of PAR4 and TFF2 with clinical pathological data of colorectal cancer.

			PAR4(TFF2) mRNA level				
		Total	increased		Not increased		*p*
		*n* = 38	*n* = 22(22)		*n* = 16(16)		
		No.	No.	%	No.	%	
Gender							
	Male	23	13(13)	57(57)	10(10)	43(43)	1.000(1.000)
	Female	15	9(9)	60(60)	6(6)	40(40)	
Age (year)							
	<60	12	7(7)	58(58)	5(5)	42(42)	1.000(1.000)
	≥60	26	15(15)	58(58)	11(11)	42(42)	
Hematogeneous metasasis							
	Negative	30	16(17)	53(57)	14(13)	47(43)	0.426(1.000)
	Positive	8	6(5)	75(63)	2(3)	25(27)	
TNM stages							
	T1 + T2	5	2(2)	40(40)	3(3)	60(60)	0.632(0.632)
	T3 + T4	33	20(20)	61(61)	13(13)	39(39)	
Lymph node metastasis							
	Negative	24	10(9)	42(38)	14(15)	58(62)	0.016(0.002)
	Positive	14	12(13)	86(93)	2(1)	14(7)	
Differentiation							
	Poor	16	14(13)	88(81)	2(3)	12(19)	0.002(0.020)
	Well and moderated	22	8(9)	36(46)	14(13)	64(54)	

**Table 2:** Association between the protein levels of PAR4 and TFF2 with clinic-pathological data of colorectal cancer.

			PAR4(TFF2) protein level				
		Total	Increased		Not increased		
		*n* = 66	*n* = 57(46)		*n* = 9(20)		*p*
		No.	No.	%	No.	%	
Gender							
	Male	35	31(26)	89(74)	4(9)	11(26)	0.724(0.389)
	Female	31	26(20)	84(65)	5(11)	16(35)	
Age (year)							
	<60	27	24(18)	89(67)	3(9)	11(33)	0.727(0.656)
	≥60	39	33(28)	85(72)	6(11)	15(28)	
Hematogeneous metastasis							
	Negative	57	48(40)	84(70)	9(17)	16(30)	0.341(1.000)
	Positive	9	9(6)	100(67)	0(3)	0(33)	
TNM stage							
	T1 + T2	12	9(9)	75(75)	3(3)	25(25)	0.347(0.743)
	T3 + T4	54	48(37)	89(69)	6(17)	11(31)	
Lymph node metastasis							
	Negative	29	26(18)	90(62)	3(11)	10(38)	0.0.720(0.233)
	Positive	37	31(28)	84(76)	6(9)	16(24)	
Differentiation							
	Poor	14	9(6)	64(43)	5(8)	36(57)	0.017(0.022)
	Well and moderated	52	48(40)	90(77)	4(12)	10(23)	
Tumor size							
	≤3cm	9	8(6)	89(67)	1(3)	11(33)	0.341(1.000)
	>3cm	57	49(40)	86(70)	8(17)	14(30)	

## Results

### The expression levels of PAR4 and TFF2 mRNA increased in colorectal cancer tissues

**Fig 1:**
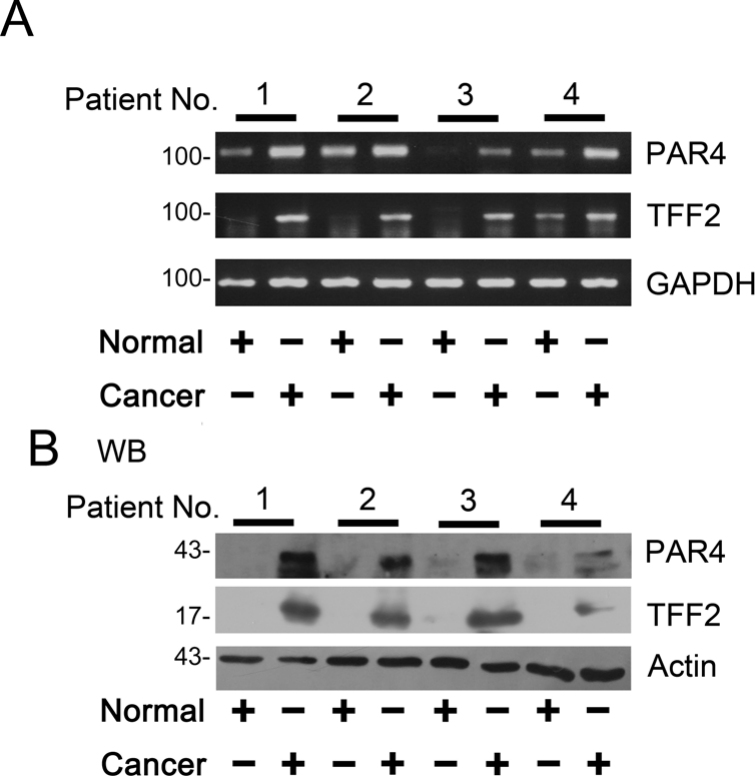
Expression of PAR4 and TFF2 in colorectal cancer tissues. (A) The matched normal (normal) and cancerous (cancer) tissues from each patients selected randomly were analyzed by RT-PCR using PAR4- and GAPDH- specific primers (n = 4). After the samples were normalized to GAPDH levels, mRNA levels of PAR4 and TFF2 were significantly increased in cancer compared to the normal tissues; (B) Western blot of tissue lysates from four cases of colorectal cancer (cancer) and relevant adjacent non-neoplastic mucosa (normal). The expression of actin was served as a control. The significant up-regulation expression of PAR4 was observed in the cancerous tissues in contrast to their matched normal tissues (n = 4).

The expression of PAR4 and TFF2 mRNA in colorectal tissues were examined by RT-PCR. As shown in Fig 1A, RT-PCR was performed on matched normal and cancer specimens randomly selected from four patients and the results showed PAR4 and TFF2 mRNA levels significantly increased in cancers tissues when compared to the matched normal tissues.

**Fig 2:**
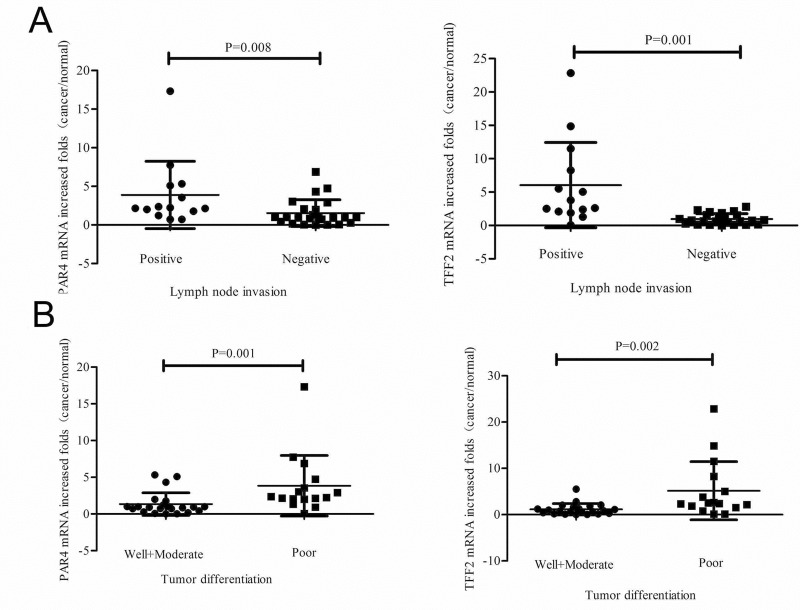
Expression of PAR4 and TFF2 mRNA in colorectal cancers and non-neoplastic tissues. Expression of PAR4 and TFF2 was measured in 38 colorectal cancer patients by real-time PCR. (A) PAR4 mRNA was increased in 86% (12 of 14) lymph node invasive tumors and 42% (10 of 24) non-invasive tumors. TFF2 mRNA was increased in 93% (13 of 14) lymph node invasive tumors and 38% (9 of 24) non-invasive tumors. There was significant difference of PAR4 (p = 0.008) and TFF2 (p = 0.001) mRNA expression between lymph node invasive tumors and non-invasive tumors; (B) PAR4 mRNA was increased in 88% (14 of 16) poor-differentiated cancers and 36% (8 of 22) well-/moderated-differentiated cancers. TFF2 mRNA was increased in 81% (13 of 16) poor-differentiated cancers and 46% (9 of 22) well-/moderated-differentiated cancers. Also, there is a significant difference of PAR4 (p = 0.001) and TFF2 (p = 0.002) mRNA expression between poor-differentiated and well-/moderated-differentiated cancers; Mean fold increase in the tumor tissue in relative to non-neoplastic colorectal tissue was shown. Median (quartile 25, quartile 75) was used to compare folds of PAR4 (TFF2) increase in lymph node invasive tumors and non-invasive tumors, and poor-differentiated cancers and well-/moderated-differentiated cancers. The increased folds of “>1” were defined as “increased”, whereas increased folds of “≤1” were defined as “not increased”.

Next, we examined the levels of PAR4 and TFF2 mRNA in 38 colorectal cancer samples by real-time PCR. The up-regulated of PAR4 and TFF2 in colorectal tumors were 58% (22 of 38) when compared with the matched non-neoplastic mucosa. We further examined the clinical significance of up-regulated expression on clinic pathological data. There was significant differences of PAR4 and TFF2 mRNA expression in lymph node invasive tumors versus non-invasive tumors (*p* = 0.016 and *p* = 0.002, chi squared text). The difference was also observed in poor-differentiated tumor versus well-/moderated- differentiated tumors (*p* = 0.002 and *p* = 0.020, chi squared text) (Table 1). In details, PAR4 mRNA was increased by 2.2 (1.8, 4.7) (quartile 25, quartile 75) folds in 14 lymph node invasive tumors and by 1.0 (0.4, 2.0) folds in 24 non-invasive tumors (*p* = 0.008, Mann-Whitney U Text); TFF2 mRNA was increased by 3.2 (2.2, 7.6) folds in 14 lymph node invasive tumors and by 0.8 (0.3, 1.5) folds in 24 non-invasive tumors (*p* = 0.001, Mann-Whitney U Text) (Fig 2A). Moreover, PAR4 mRNA was increased by 2.3 (2.0, 3.8) folds in 16 poor-differentiated tumors and by 0.9 (0.5, 1.2) folds in 22 well-/moderated- differentiated cancers (*p* = 0.001, Mann-Whitney U Text); TFF2 mRNA was increased by 2.2 (1.8, 5.8) folds in 16 poor- differentiated tumors and by 0.8 (0.3, 1.5) folds in 22 well-/moderated-differentiated cancers (*p* = 0.002, Mann-Whitney U Text) (Fig 2B).

### Protein levels of PAR4 and TFF2 were increased in the colorectal cancer tissues

PAR4 and TFF2 proteins were low or undetected in the normal colorectal mucosa by western blotting analysis (Fig 1B). However, in patient tissues with colorectal cancer, after the samples were normalized to β-actin levels, a significant increased expression of PAR4 and TFF2 was observed in colorectal cancer tissues as compared with the matched nonmalignant tissues (Fig 1B).

**Fig 3:**
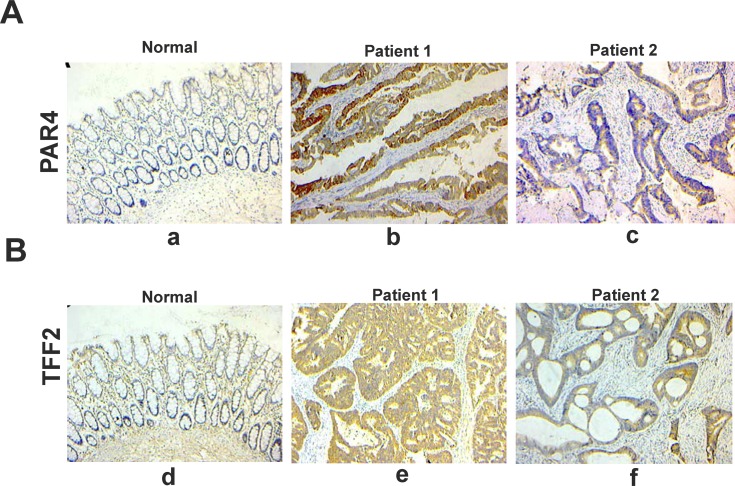
Representative photomicrographs of immunohistochemical staining of PAR4 and TFF2 in paraffin sections of colorectal tissues. (A) immunohistochemical staining for PAR4. (a) normal colorectal mucosa; (b) colorectal cancer tissue of number one patient. (c) colorectal cancer tissue of number two patient; (B) immunohistochemical staining for TFF2. (d) normal colorectal mucosa; (e) colorectal cancer tissue of number one patient; (f) colorectal cancer tissue of number two patient. Bar, 100 μm for each panel, and 50 μm for insets.

Next, we performed immunohistochemical staining to analyze the protein expression levels of PAR4 and TFF2 *in vivo*. There was nearly no expression of PAR4 and TFF2 in non-neoplastic colorectal epithelial cells, but the expression was significantly increased in malignant colorectal epithelial cells. Furthermore, the majority of PAR4 and TFF2 staining in malignant colorectal cancer samples were localized in the cytoplasm (Fig 3). In 66 analyzed samples, PAR4 expression was up-regulated in 57 (86.1%) colorectal cancer samples when compared with the matched normal mucosa. TFF2 expression was up-regulated in 46 (69.7%) colorectal cancer samples. In addition, the differences in PAR4 and TFF2 protein expression between poor-differentiated and well-/moderated- differentiated tumors were significant (*p* = 0.017 and *p* = 0.022, chi squared text) (Table 2).

### Overexpression of PAR4 increased invasion activity of LoVo cells induced by TFF2

**Fig 4:**
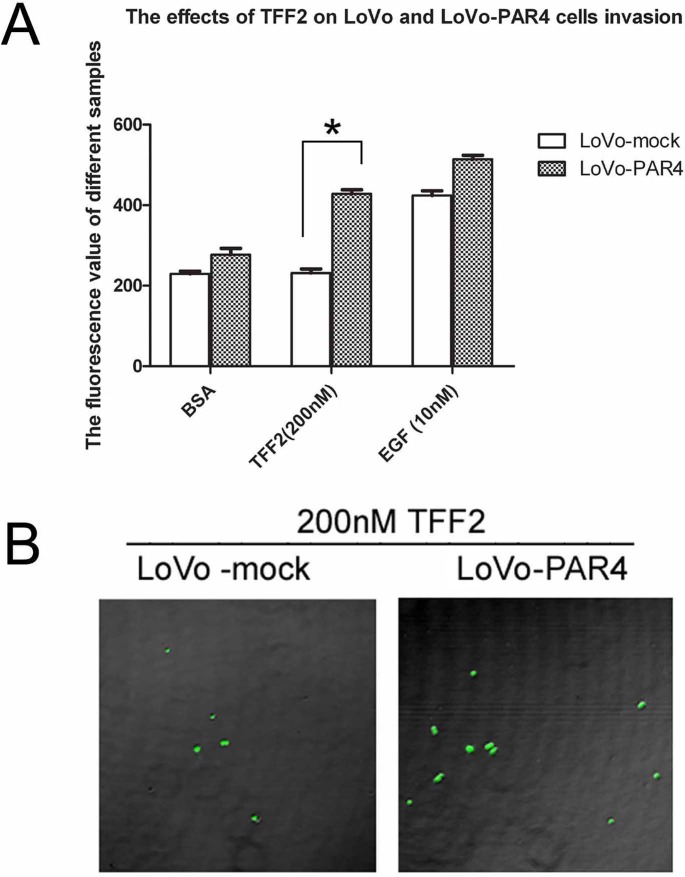
Effect of recombinant TFF2 on the invasion of LoVo-mock and LoVo-PAR4 cells. (A) Cell invasion stimulated by TFF2 was tested using an InnoCyte cell invasion assay. Invasion of LoVo-mock and LoVo-PAR4 cells stimulated by 200nM TFF2 was tested, with BSA and 10nM EGF as controls. The results showed significant difference of 200nM TFF2 on LoVo-mock and LoVo-PAR4 cells invasion activity. Data were presented as means ± SD of three independent experiments. *p<0.05; (B) Invasion of LoVo-mock and LoVo-PAR4 cells stimulated by 200nM TFF2 were investigated by Confnocal laser fluorescence microscopy.

Our previous results have showed that TFF2 promotes cell migration via PAR4 [17]. To investigate the role of PAR4 in TFF2-induced cell invasion, we examined the effect of TFF2 on LoVo-mock and LoVo-PAR4 cells invasion activities. As shown in Fig 4A, 200 nM of TFF2 did not induce invasion activity in LoVo-mock cells by Matrigel assay. However, it significantly increased the invasion activity in LoVo-PAR4 cells. The confocal microscopy also showed that the invasion activity of LoVo-PAR4 cells was 2 folds higher than LoVo-mock cells when stimulated by TFF2 (Fig 4B).

### PAR4 Knockdown in HT-29 cells decreased cell invasion activity in respond to TFF2

**Fig 5:**
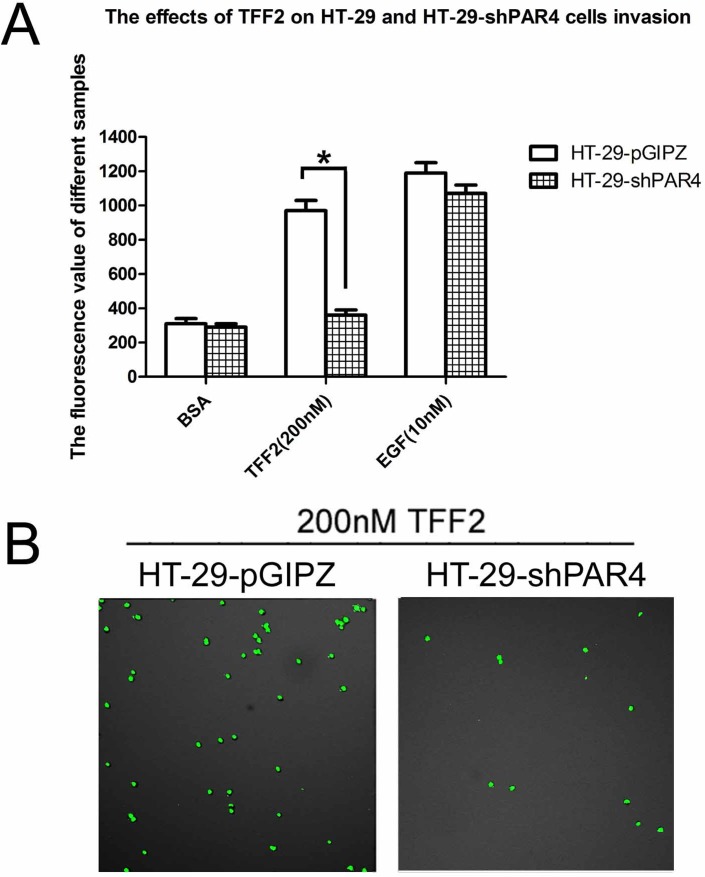
Effect of recombinant TFF2 on cell invasion of PAR4-knockdowned HT-29 cells. (A) Cell invasion stimulated by TFF2 was tested using an InnoCyte cell invasion assay. Invasion activity of HT-29-pGIPZ and HT-29-shPAR4 cells were treated with 200 nM TFF2 to assess the invasion activity, BSA and 10nM EGF were used as controls. There was significant difference of 200nM TFF2 on HT-29-pGIPZ and HT-29-shPAR4 cells invasion. Data were presented as means ± SD of three independent experiments, *p<0.05; (B) Invasion cells of HT-29-pGIPZ and HT-29-shPAR4 stimulated by 200nM TFF2 were investigated by Confnocal laser fluorescence microscopy.

We next determined the effect of TFF2 on cells invasion activity in the PAR4- knockdowned HT-29 cells. As shown in Fig 5A, 200 nM TFF2 significantly increased the invasion activity of HT-29-pGIPZ cells by Matrigel assay, but had no effect on the PAR4-knockdowned cells. Confocal microscopy results also showed that the invasion of HT-29-pGIPZ is more than 3–4 folds of HT-29-shPAR4 cells when the cells are stimulated by TFF2 (Fig 5B). The results suggested that TFF2-induced invasion was PAR4-dependent.

### Restoration of PAR4 expression by treatment of 5-Aza-doxy in colorectal LoVo cells

**Fig 6:**
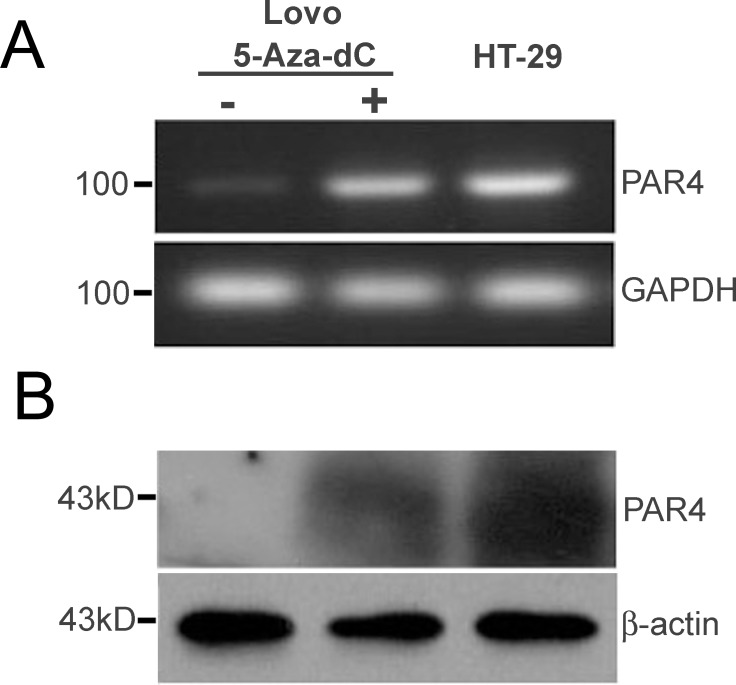
5-Aza-dC induced PAR4 expression in colorectal cancer cell, LoVo. LoVo, a non- PAR4 expressing cells, were incubated with 5-Aza-dC (10 μM) or DMSO for 72 hours, and the cells were used to extract mRNA and protein. (A) The expression level of PAR4 mRNA was examined by RT-PCR; (B) The expression level of PAR4 protein was examined by western blot. The colorectal cancer cell line HT-29 expressed PAR4 was used as a positive control.

As reported in Zhang’s research paper, PAR4 was not expressed in human colon cancer cell line **of** LoVo [17]. To elucidate the potential molecular mechanism underlying PAR4 up-regulated expression in colorectal cancer tissues, the LoVo cells were treated with 5-Aza-dC, a demethylating agent. In Fig 6A and 6B, RT-PCR and Western blotting showed that the expression level of PAR4 was restored after 3 days of treatment with 5-Aza-dC, and human colorectal cancer cell lines HT-29 expressing PAR4 was used as a control.

### Analysis methylation level of the promoter region of PAR4 in colorectal cancer tissues

**Fig 7:**
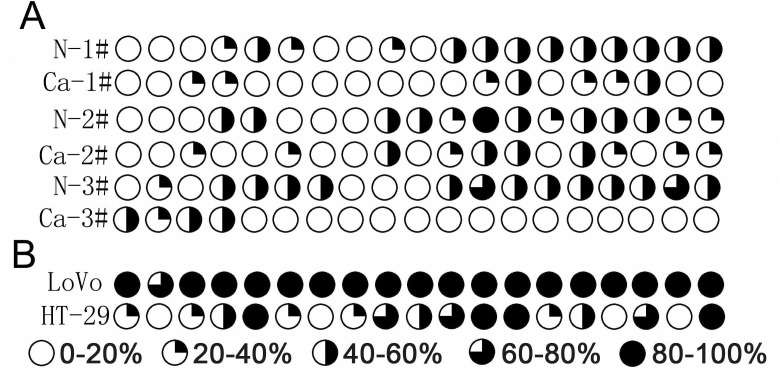
Genomic bisulfite sequencing of CpG methylation sites of PAR4 promoter in colorectal cancer tissues and cell lines. (A) PAR4 promoter methylation was analyzed in DNA from three colorectal cancer and their matched non-neoplastic tissues. (B) PAR4 promoter methylation in DNA from colorectal cancer cells lines, LoVo and HT-29. Average methylation at each analyzed CpG site in the PAR4 promoter is indicated based on bisulfite sequencing of 19 individual clones.

Because demethylation with 5-Aza-dC leads to the restoration of PAR4 expression in LoVo cells, we compared the methylation level of PAR4 promoter between colorectal cancer and the matched normal tissues. Using the genomic bisulfite sequencing method, we examined 19 CpG sites in a 380 bp region of the PAR4 promoter. Three colorectal cancer samples with high PAR4 expression displayed pronounced hypomethylations, and their average methylation rates of 19 CpG sites is 22.1%. However, the hypermethylation was found in matched non-cancer tissues which had low expression of PAR4, and their average methylation rates was 41.6% (Fig 7A). High PAR4-expression HT-29 cells displayed hypomethylation in its promote while non-PAR4 expression LoVo cells displayed hypermethylation (Fig 7B).

## Discussion

The difference expression of PAR4 and TFF2 was detected in various tumors, such as the pancreas cancer, lung cancer and gastric cancer, and this difference expression was associated with tumor cell growth, migration, invasion and angiogenesis [11,22–24]. Because PARs play important roles in various cancers and they have become attractive for the development of novel therapeutics targets[25]. In the study, we presented some fundamental data that the expression levels of PAR4 and TFF2 were significantly increased in colorectal cancer tissues when compared to the matched noncancerous tissues, especially in the metastatic positive lymph nodes and poorly differentiated tumors. The majority of PAR4 and TFF2 in colorectal cancer tissues are localized in the cytoplasm as shown by Immunostaining. The increase of PAR4 expression in colorectal cancer was consistent with Gratio’s research results [12], in which PAR4 was absent in normal colonic mucosa and epithelial cells, but high in colon adenocarcinoma tissues, and 71% human colorectal cell lines show strong immunostaining of PAR4 [12]. On the contrary, PAR4 expression was decreased in gastric cancer, esophageal squamous cancer and lung adenocarcinoma tissues comparing with the relative normal mucosa [17,26,27]. The results indicated that the function of PAR4 was different in tumor progressions. Since the unknown factors might be involved in the aberrant expression of PAR4, there are much works need to understand the mechanism of PAR4 regulation before it can be a potential target drug for cancer therapy.

TFF2, as a principal cytoprotective trefoil factor, was mainly expressed in stomach, and the expression was dysregulated during gastric cancer progression [16,23,28]. In Jiang’s study, TFF2 expression was markedly decreased in gastric cancer, suggesting the role of TFF2 as a tumor suppressor in gastric carcinogenesis and metastasis [29]. In colon mucosa, TFF2 was expressed with low intensity, but the expression of TFF2 in the progress of colorectal cancer was not clear [30]. In our research, it was interesting to find TFF2 expression was also increased in cancer tissues when compared to the relative normal colorectal mucosa. Furthermor, our results revealed that the recombinant human TFF2 could activate PAR4 to promote LoVo-PAR4 and HT-29-pGIPZ cell invasion activity, but had no significant invasion effect on LoVo-mock and HT-29-shPAR4 cells. The results consist with our previous finding that TFF2 activated PAR4 to promote epithelial cell migration via ERK1/2 phosphorylation [17]. Phosphorylation of ERK1/2 is required for cell migration and is central to TFF mediated signaling [14,31]. Hence the facts that the up-regulation expression of PAR4 and TFF2 in colorectal cancer, their co-localized cytoplasm expression patters, and TFF2 promoting cell migration and invasion via PAR4, suggesting the interaction of PAR4 and TFF2 *in vivo* and *in vitro* via the unknown mechanism to be further investigated.

Transcriptional silencing by promoter hypermethylation has recently emerged as one of the important mechanisms in gastric cancer development [32]. In this study, we found that the 5-Aza-dC, a demethylating agent, restored PAR4 expression in LoVo cells. We further analyzed the methylation status of cytosine in CpG dinucleotide located within non-CpG islands at the PAR4 promoter region by using colorectal cancer tissues and cell lines with different PAR4 expression level. Promoter hypermethylation was confirmed in normal colorectal mucosa with low expression of PAR4. However, promoter hypomethylation was found in colorectal cancer tissues with high expression of PAR4. The results indicated that promoter hypomethylation may promote the transcription of PAR4. Zhang et al found that loss of PAR4 expression in gastric cancers may result from hypermethylation of the PAR4 promoter [33]. But PAR4 decreased expression in lung adenocarcinoma was not related to promoter methylation [2727]. These facts suggested that promoter methylation level was only one factor leading to the difference of PAR4 expression, and its expression was regulated by multiple factors.

In conclusion, the research showed that PAR4 and TFF2 expression were frequently up-regulated in colorectal cancer and the increased expression was associated with the clinically aggressive phenotype. DNA hypomethylation leads to the up-regulated expression of PAR4 in colorectal cancer tissues. We also showed PAR4 promoted colorectal cancer cells’ invasion in respond to TFF2. These results will help us understanding molecular mechanism of TFFs and PARs in carcinogenesis and progression of colorectal cancers.
